# Langzeitverläufe der Alkoholabhängigkeit

**DOI:** 10.1007/s00115-024-01719-0

**Published:** 2024-08-21

**Authors:** Ulrich John, Hans-Jürgen Rumpf, Sabine Hoffmann, Christian Meyer, Falk Kiefer

**Affiliations:** 1 Institut für Community Medicine, Abteilung für Präventionsforschung und Sozialmedizin, Universitätsmedizin Greifswald, Greifswald, Deutschland; 2 Klinik für Psychiatrie und Psychotherapie, Universität zu Lübeck, Lübeck, Deutschland; 3 Zentralinstitut für Seelische Gesundheit, Klinik für Abhängiges Verhalten und Suchtmedizin, Mannheim, Deutschland; 4 Institut für Community Medicine, Abteilung für Präventionsforschung und Sozialmedizin, Universitätsmedizin Greifswald, Walther-Rathenau-Str. 48, 17475 Greifswald, Deutschland

**Keywords:** Nikotinabhängigkeit, Therapieinanspruchnahme, Mortalität, Frühintervention, Lebensdauer, Nicotine dependence, Utilization of treatment, Mortality, Early intervention, Duration of life

## Abstract

**Hintergrund:**

Langzeitbetrachtungen der Alkoholabhängigkeit können Aufschluss zu Ätiologie, Prävention und therapeutischer Versorgung bieten.

**Ziel:**

Beschreibung empirischer Befunde zur Entwicklung in eine Alkoholabhängigkeit hinein und Entwicklungen aus ihr heraus.

**Methodik:**

Narrative Literaturrecherche, Analyse von Ergebnissen aus Kohortenstudien in der Bevölkerung.

**Ergebnisse:**

Risikofaktoren lassen eine erhöhte Wahrscheinlichkeit der Ausbildung einer Alkoholabhängigkeit schätzen. Jugendliche zeigten innerhalb von 8 Jahren nach Beginn des Alkoholkonsums Symptome einer Alkoholabhängigkeit. Besteht diese, ist mit einer um 17,6 Jahre verkürzten Lebensdauer zu rechnen. Daten einer Bevölkerungsstichprobe zeigten gegenüber Menschen ohne psychische Störung ein 2,8fach erhöhtes Risiko, vorzeitig zu versterben. Die Schwere der Alkoholabhängigkeit erwies sich als Prädiktor vorzeitigen Versterbens. Nikotinabhängigkeit kann neben der Alkoholabhängigkeit zur verkürzten Lebensdauer beitragen. In einer Bevölkerungsstichprobe hatten 90,2 % der alkoholabhängigen Personen an keiner qualifizierten Entzugsbehandlung und 78,4 % an keiner Entzugsbehandlung in einer psychiatrischen Einrichtung teilgenommen. Remissionen ohne suchtspezifische Hilfe sind nachgewiesen und stellen den überwiegenden Remissionsweg dar.

**Diskussion:**

Zur Reduktion der ungünstigen langfristigen Verläufe sollten Prävention und Suchtkrankenversorgung mehr als bisher auf den Bedarf in der Bevölkerung ausgerichtet werden. In der psychiatrischen und weiteren medizinischen Praxis sollten leitliniengemäß Screenings auf Alkoholgebrauchsstörungen und entsprechende Kurzinterventionen durchgeführt werden.

## Abstract (en)

**Background:**

Descriptions of long-term histories of alcohol dependence provide insight into the etiology, prevention and care.

**Objective:**

Report of empirical findings about the development into and remission from alcohol dependence.

**Method:**

Narrative literature review, analysis of findings from cohort studies in the general population.

**Results:**

Risk factors provide the opportunity to estimate increased probabilities of developing an alcohol dependence. Adolescents disclosed symptoms of alcohol dependence within 8 years after the first alcohol consumption. Alcohol dependence is related to a life expectancy 17.6 years fewer than in the general population. Data of a general population sample revealed a risk of early death 2.8 times higher than among people without mental disorders. The severity of alcohol dependence was shown to be a predictor of premature death. Nicotine dependence can add to the shortening of life in addition to alcohol dependence. Among the alcohol dependent residents in a general population sample, 90.2% did not have utilized detoxification treatment that included motivational treatment and 78.4% did not have utilized standard detoxification treatment in a psychiatric treatment facility. Remission without formal help has been proven. It is the main route of remission.

**Conclusion:**

To reduce unfavorable courses of alcohol dependence, prevention and treatment should be focused more on the needs in the general population. In psychiatric and other medical routine care, screening and an appropriate brief intervention should be carried out.

Langzeitverläufe der Alkoholabhängigkeit zu beschreiben, bedeutet im Idealfall Lebensläufe über mehr als eine Generation hinweg zu analysieren. Geliefert hat die Forschung überwiegend Ergebnisse zu Abschnitten im Leben alkoholabhängiger Menschen. Was Langzeit bei der Alkoholabhängigkeit bedeutet, hängt von den Erkenntnisinteressen ab. Bei ätiologischen Fragestellungen ist das die Entwicklung in die Abhängigkeit hinein, bei Fragen der Optimierung von Hilfe die Entwicklung aus ihr heraus. Wenn es um die Prädiktion von Morbidität und Mortalität geht, sind Jahrzehnte des Lebens einschlägig. Ziel dieser Arbeit ist, erstens einen Überblick über Untersuchungsergebnisse zu Langzeitverläufen der Alkoholabhängigkeit mit der Entwicklung in eine Alkoholabhängigkeit hinein und aus ihr heraus zu geben. Zweitens soll gezeigt werden, was daraus für die Planung von Prävention und Suchtkrankenversorgung folgt.

## Entwicklung in eine Alkoholabhängigkeit hinein

Die Wahrscheinlichkeit, dass Personen in der Bevölkerung eine Alkoholabhängigkeit entwickeln, lässt sich mit Risikofaktoren schätzen. Dazu zählen genetische Faktoren, Alkoholabhängigkeit in der Elternfamilie, männliches Geschlecht, früher Beginn des Alkoholkonsums und Umgang mit Alkohol in der sozialen Umgebung im Jugendalter [22]. Hinzu kommen Trinkmengen im weiteren sozialen Umfeld und in der Nation.

Ein Teil des Wissens stammt aus Studien an Familien alkoholabhängiger Erwachsener und ihren Kindern [1] sowie aus einer Bevölkerungsstichprobe Jugendlicher und junger Erwachsener, die bei der Entwicklung über ca. 8 Jahre begleitet wurden [4]. Die empirischen Untersuchungen legen nahe, dass Eltern das Risiko einer Alkoholabhängigkeit über Erbfaktoren und Verhalten an ihre Kinder weitergeben [30]. Die Zahl der vom Vater erfüllten Kriterien einer Alkoholabhängigkeit waren mit Risikokonsum der Kinder in ihrem Jugendalter verknüpft [29]. Personen mit einem alkoholabhängigen Elternteil hatten im Vergleich zu Personen ohne alkoholabhängigen Elternteil einen höheren Polygen-Risikoscore [18]. Je größer sein Wert war, desto mehr Kriterien einer Alkoholabhängigkeit erfüllten die Kinder [18].

Polygen-Risikoscores bieten – anders als die Alkoholabhängigkeit bei Eltern – einen besonders wenig verzerrten Schätzer des genetischen Risikos [18]. Wenngleich sie bisher nicht für die Anwendung im individuellen Fall empfohlen sind, legt die Evidenz nahe, dass sie zur Prädiktion einer Alkoholabhängigkeit durch männliches Geschlecht, komorbide psychische Störungen, Alter bei erstem Alkoholkonsum und Alkoholkonsum in der Gruppe gleichaltriger Jugendlicher beitragen können [22].

### Entwicklung von Jugendlichen

Eine Stichprobe von 3021 Personen im Alter von 14 bis 24 Jahren wurde per Zufall aus der Bevölkerung einer Region Deutschlands ausgewählt und mit dem Composite International Diagnostic Interview (M-CIDI) befragt [2, 3]. Acht Jahre später wiesen 13,1 % das Alkoholabhängigkeitssymptom der Toleranzbildung, 5,0 % viel Zeit für Aktivitäten um den Alkoholkonsum, 3,4 % Kontrollverlust und 0,6 % Entzugssymptome auf [3]. Von den Symptomen einer Alkoholabhängigkeit traten bereits im ersten Jahr nach Beginn des Konsums von Alkohol auf: 10 % der Nennungen von Kontrollverlust und 5 % der Nennungen von Entzugssymptomen [3]. Psychische Störungen, besonders Nikotinabhängigkeit, bilden der Studie zufolge Risikofaktoren für die Ausbildung einer Alkoholabhängigkeit [4].

## Entwicklung ab Bestehen einer Alkoholabhängigkeit

Drei Wege der Kontaktaufnahme mit alkoholabhängigen Menschen wurden beschritten:

1. Ziehung und Kontaktierung einer Bevölkerungsstichprobe,
2. Nutzung von Medien oder Institutionen außerhalb der Medizin, u. a. Militär,
3. Suchttherapieeinrichtungen.

Per Zufall ausgewählte Stichproben der Allgemeinbevölkerung ermöglichen, dass der Grad der Verzerrung von Ergebnissen durch Selektivität der Stichprobe besonders gering ist.

Betrachtbare Endpunkte der Verläufe sind Merkmale der Alkoholabhängigkeit, Alkoholkonsum und -abstinenz, darüber hinaus Kokonsum weiterer Suchtmittel, zusätzliche Krankheiten, Lebensqualität und die Überlebensdauer. Sie hat bei der Beurteilung von Langzeitverläufen den herausragenden Stellenwert. Alkoholabhängigkeit ist durch reduzierte Überlebenszeit gekennzeichnet. Sie zu erhöhen, sollte das primäre Ziel der Interventionen sein. Alle Bemühungen um eine gute Versorgung in Prävention und Medizin sollten auf eine Verlängerung der Lebenszeit bei möglichst hoher Lebensqualität ausgerichtet werden.

### Zufallsstichproben der Bevölkerung

Wir unterscheiden im Folgenden frühere und gegenwärtige Alkoholabhängigkeit und orientieren uns dabei an den Kriterien internationaler Klassifikationen der Erkrankungen. Bei einer gegenwärtigen Alkoholabhängigkeit bestanden die Kriterien in den letzten 12 Monaten vor der Diagnosestellung, bei früherer oder remittierter Alkoholabhängigkeit im Zeitraum vor, nicht jedoch in den letzten 12 Monaten. Wir konzentrieren uns auf die Alkoholabhängigkeit selbst und aus psychiatrischer Sicht naheliegende Koexistenz von Nikotinabhängigkeit, weitere psychiatrische Komorbidität und weitere mit der Alkoholabhängigkeit in Zusammenhang stehende verhaltensbedingte Gesundheitsrisiken. Somatische Störungen, die im Verlauf der Alkoholabhängigkeit entstehen, lassen wir außer Acht [26, 27].

### Lebenszeit bei Alkoholabhängigkeit

Die Mortalitätsstatistik Deutschlands zeigt für das Jahr 2022 ein durchschnittliches Sterbealter von 61,6 Jahren (Frauen 64,2, Männer 60,8 Jahre), wenn die Todesbescheinigung eine Alkoholabhängigkeit (F10.2 Alkoholabhängigkeitssyndrom) ausweist [28]. Das entspricht 17,6 Lebensjahren weniger als in der Gesamtbevölkerung (Frauen 17,7, Männer 15,6 Jahre; [28]). Es gibt kaum eine Krankheit, die mit einem so sehr erniedrigten Sterbealter einhergeht. Die beiden größten Todesursachengruppen, Krebs- und Herz-Kreislauf-Erkrankungen, weisen jeweils ein höheres Sterbealter auf. Möglicherweise wird Alkoholabhängigkeit in den zugrunde liegenden Todesbescheinigungen nur dann dokumentiert, wenn sie als Todesursache besonders offensichtlich ist.

Die denkbare Unterschätzung der Alkoholabhängigkeit in der Todesursachenstatistik lässt sich mit Kohortenstudien verringern. In der Zufallsstichprobe einer regionalen Bevölkerung Deutschlands im Alter von 18 bis 64 Jahren wurde eine psychiatrische Diagnostik mit dem Composite International Diagnostic Interview bei 4075 Personen durchgeführt und ausgewertet (Studie „Transitions in Alcohol Consumption and Smoking“ [TACOS]; [21]). Das waren 69,9 % der infrage kommenden Personen der Stichprobe. Von den Studienteilnehmerinnen mit einer gegenwärtigen Alkoholabhängigkeit starben im Laufe von 20 Jahren 36,7 %. Von den Studienteilnehmern waren es 30,3 %, von den alkoholabhängigen Personen zusammen 31,6 % gegenüber 14,2 % in der Gesamtstichprobe [13]. Die Wahrscheinlichkeit vorzeitigen Versterbens im Vergleich zu Personen ohne psychische Störung war 2,83fach höher (95 %-Konfidenzintervall: 2,08–3,86; [13]). Dieser Wert war über das Alter hinaus adjustiert für Geschlecht und mögliche weitere psychische Störungen (weitere Substanzstörungen, Major-Depression, Dysthymia, bipolare Störungen, Angststörungen, Panikstörungen, Zwangsstörungen, somatoforme Störungen, Schmerzstörungen, Essstörungen). Eine frühere Alkoholabhängigkeit prädizierte die verbleibende Lebenszeit ähnlich wie eine gegenwärtige Alkoholabhängigkeit [14]. Für Alkoholmissbrauch ergab sich keine erhöhte Mortalität. Frühere Bevölkerungsstudien hatten Alkoholabhängigkeit gemeinsam mit Alkoholmissbrauch untersucht und fanden dementsprechend niedrigere Sterbewahrscheinlichkeiten [8, 19].

Die Schwere der Alkoholabhängigkeit erwies sich als Prädiktor für frühen Tod bei Alkoholabhängigen [14]. Gleichgültig ob die Zahl der Abhängigkeitskriterien nach dem Diagnostischen und Statistischen Manual Psychischer Störungen oder der Wert einer standardisierten Skala der Schwere der Abhängigkeit zugrunde gelegt wurde: Je höher dieser Wert oder die Zahl der Abhängigkeitskriterien war, desto weniger Lebensjahre verblieben für die betroffenen Personen [14]. Mit jedem erfüllten Kriterium der Alkoholabhängigkeit war die Wahrscheinlichkeit frühen Todes um 25 % höher als bei den Personen, die kein Symptom einer Alkoholabhängigkeit zeigten [14].

In der Bevölkerungsstichprobe war die Alkoholabhängigkeit bei Patient*innen nach Suchttherapie besonders schwer ausgeprägt im Vergleich zu denjenigen, die weder eine Entwöhnungs- noch eine Entzugsbehandlung in Anspruch genommen hatten [14]. Diese lebten auch länger als die alkoholabhängigen Patient*innen nach Behandlung [14]. Unter den alkoholabhängigen Personen, die weder an einer Entwöhnungs- noch einer Entzugsbehandlung teilgenommen hatten, waren 24,3 %, unter denen nach Entwöhnungsbehandlung 47,4 %, unter denen nach ausschließlich einer Entzugsbehandlung 66,67 % verstorben [14]. Von den Studienteilnehmer*innen mit der Lebenszeitdiagnose einer Alkoholabhängigkeit hatten 81,0 % an keiner Entwöhnungsbehandlung, 90,2 % an keiner qualifizierten Entzugsbehandlung in einer psychiatrischen Einrichtung und 78,4 % an keiner Entzugsbehandlung in einer psychiatrischen Einrichtung teilgenommen [23]. Eine mögliche Schlussfolgerung lautet, dass unser bisheriges Behandlungssystem nur eine Minorität schwer betroffener Patient*innen erreicht. Dabei gaben 92,7 % an, in den letzten 12 Monaten vor der Befragung Kontakt zu einer Arztpraxis oder einer anderen medizinischen Einrichtung gehabt zu haben [23].

### Komorbidität mit Nikotinabhängigkeit

Besonders bedeutsam in Bezug auf die Überlebenszeit ist das gleichzeitige Bestehen einer Alkohol- und einer Nikotinabhängigkeit. Daten aus Suchttherapieeinrichtungen zeigen, dass mehr als 75 % der alkoholabhängigen Patient*innen auch Tabak rauchten [12]. In der TACOS-Stichprobe waren unter den gegenwärtigen Alkoholabhängigen 77,8 % gleichzeitig Raucher*innen [15]. Die Personen mit der höchsten Zahl erfüllter Kriterien einer Alkoholabhängigkeit umfassten auch den höchsten Anteil gegenwärtig Nikotinabhängiger (60,0 %) sowie den höchsten Anteil von Raucher*innen (86,7 %; [15]). Die Ergebnisse legen nahe, dass sowohl die Alkoholabhängigkeit als auch die Nikotinabhängigkeit zum vorzeitigen Versterben beitragen [15]. Unter remittierten Alkoholabhängigen hatten 29,6 % eine gegenwärtige Nikotinabhängigkeit [15]. In dieser Personengruppe war eine niedrige Lebenserwartung besonders wahrscheinlich im Vergleich zu Personen mit geringem bis moderatem Alkoholkonsum, die im Leben zuvor nicht geraucht hatten (Hazard Ratio 4,55; 95 %-Konfidenzintervall 2,38–8,70; [15]). Ihnen gegenüber wiesen selbst diejenigen mit früherer Alkohol- und zusätzlich früherer Nikotinabhängigkeit eine erhöhte Wahrscheinlichkeit für geringe Lebenserwartung auf (Hazard Ratio 2,81; 95 %-Konfidenzintervall 1,33–5,94; [15]).

Die Befunde legen zwei Schlüsse nahe: Eine Remission bei Alkohol- oder Nikotinabhängigkeit mildert die Reduktion von Lebenszeit lediglich ab, beide Krankheiten tragen ähnlich stark zum Versterben bei. Bei Alkoholabhängigen sind über das Tabakrauchen hinaus weitere Belastungen durch Bewegungsmangel und Ernährungsdefizite zu erwarten. Auch sie sind an der Prädiktion vorzeitigen Versterbens beteiligt [16].

### Studien mit weiteren Kontaktaufnahmen

Menschen, die angeben, Alkoholprobleme gelöst zu haben, wurden mittels Anzeigen oder Artikel in Medien gesucht und mit denen verglichen, die Suchthilfen in Anspruch nahmen. Unter Alkoholabhängigen, die durch Medienaufrufe und zum kleineren Teil aus einer Bevölkerungsstichprobe gewonnen worden waren, blieben 92,3 % zwei Jahre oder länger remittiert [24]. Sie hatten keine suchtspezifische Hilfe in Anspruch genommen, die über zwei Selbsthilfegruppenbesuche hinausging. Bedeutsam sind bei dieser Remission die Schwere der Alkoholabhängigkeit, das Ausmaß alkoholbezogener Probleme und die soziale Unterstützung. Empirische Befunde zeigen, dass es unter alkoholabhängigen Menschen, die ohne suchtspezifische Hilfe remittierten, auch die mit hoher Schwere der Abhängigkeit und ausgeprägten alkoholbezogenen Problemen gibt [5]. Auch bei schwerer Symptomatik kann soziale Unterstützung zu einer Remission beitragen. Eine komorbide psychische Störung scheint keinen Einfluss auf die Remission ohne formelle Hilfe auszuüben [6].

Insgesamt zeigen empirische Ergebnisse, dass unter alkoholabhängigen Menschen, die keine Suchttherapie oder -selbsthilfegruppe in Anspruch nehmen, 66–78 % remittieren [24]. Schätzungen aus Europa, den USA und Australien zufolge ließen ca. 80 % der Personen mit einer Alkoholgebrauchsstörung diese im letzten Jahr vor der Befragung nicht behandeln [20]. Als Gründe für Remissionen ohne suchtspezifische Hilfe wurden besonders familiäre und gesundheitliche Probleme benannt. Dabei gilt zumeist als Kriterium für Remission, dass die Person 12 Monate oder länger Alkohol nicht oder in kontrollierter Weise konsumiert [24].

Aufgrund der Ergebnisse zu Remissionen ohne suchtspezifische Hilfe wurde empfohlen, Kurzinterventionen zu nutzen. Sie können helfen, frühzeitig Remissionsprozesse zu fördern [24]. Die Auseinandersetzung mit dem eigenen Alkoholkonsum sollte frühzeitig im Verlauf angeregt werden. Dazu gehören gedankliche Prozesse, etwa das Abwägen positiver und negativer Konsequenzen des Konsums [24]. Modelle der Behandlungsplanung sollten einen proaktiven Zugang zu betroffenen Menschen beinhalten. Dabei wird stets die Früherkennung und -intervention in der medizinischen Basisversorgung genannt, auf die intensivere Behandlung folgen kann [17, 20]. Darüber scheint es einen breiten Konsens zu geben. Allein die Bereitschaft zur Schaffung des erforderlichen Personals und zur Vergütung der Leistungen in der medizinischen Basisversorgung fehlt bisher [11].

### Studien mit Patient*innen aus Suchttherapieeinrichtungen

Träger von Suchttherapieeinrichtungen zeigen mit Befragungen ehemaliger Patient*innen, dass ein Teil von ihnen Alkoholabstinenz angibt. Das mögen für die Behandlung bedeutende Rückmeldungen sein. Jedoch nicht alle Patient*innen stellen Informationen bereit. In einer Untersuchung aus 21 Suchttherapieeinrichtungen in Deutschland stimmten 82 % der alkoholabhängigen Patient*innen im Alter von 17 bis 66 Jahren während der Therapie einer Befragung zu. Vier Jahre später wurden noch 1057 der 1410 Studienteilnehmer*innen für Auskünfte zum Umgang mit Alkohol erreicht. Ihnen zufolge lebten 46,4 % seit Beginn der Behandlung alkoholabstinent [9].

### Prävention und therapeutische Versorgung

Die empirische Forschung zu Langzeitverläufen der Alkoholabhängigkeit zeigt Remissionen auf unterschiedliche Art und Weise, aber auch Begrenzungen der bisherigen therapeutischen Versorgung und eine hohe Mortalität. Eine Lösung dieser Probleme ist durch Prävention und größere Vielfalt von Hilfeangeboten zu erwarten. Zur Prävention sind die notwendigen Interventionen bekannt. Sie müssen erstens Maßnahmen umfassen, Alkohol unattraktiv zu machen. Dazu zählen Preiserhöhungen, Steuerung der Erhältlichkeit alkoholischer Getränke mit Orten und Zeiten, Jugendschutz, Werbung gegen Alkohol und Verbote von Werbung für Alkohol, Alkoholabstinenz bei Teilnahme am Straßenverkehr und Förderung sozialer Normen zum Alkohol als ein gesundheitsgefährliches Produkt.

Zweitens werden Kampagnen und individualisierte Programme der Motivierung zur Alkoholkonsumreduktion benötigt. Dabei sind ganze Bevölkerungsgruppen zu kontaktieren, z. B. in der Arbeitswelt und medizinischen Versorgung. Die Motivierung zur Alkoholreduktion kann ökonomisch über Computerexpertensysteme erfolgen [7]. Suchtberatung sollte auf die Bevölkerung ausgerichtet werden mit Screenings und Kurzinterventionen [10]. Die Versorgung ist in ihrem Schwerpunkt auf einen frühen Zeitpunkt im Leben auszurichten, in dem die Person erst kurze Zeit dem Alkohol ausgesetzt ist [25]. Die Suchtkrankenversorgung und die medizinische Basisversorgung sollten Angebote schaffen, die eine Majorität der betroffenen Menschen erreichen.

## Fazit für die Praxis

- Psychiater*innen sollten bei allen Patient*innen den Alkoholkonsum proaktiv ansprechen. Es ist stets an die Möglichkeit der Entwicklung einer Alkoholabhängigkeit zu denken und zur Reduktion von Alkoholkonsum zu motivieren. Verfahren sind vorhanden.
- Es sollte stets ein Screening auf Alkoholabhängigkeit und bei positivem Ergebnis eine weiterführende Diagnostik durchgeführt werden.
- Die Nikotinabhängigkeit und das Tabakrauchen tragen zu der Verkürzung der Lebenserwartung bei und sollten mehr als bisher in Interventionen berücksichtigt werden.
- Besonders hohen Impact verspricht die Ansprache des Alkoholkonsums in der medizinischen Basisversorgung. In Zusammenarbeit mit der psychiatrischen Versorgung und der Suchtkrankenversorgung sollten Leistungen ausgebaut werden, die im Verlauf frühzeitiger als bisher einsetzen. Ein Versorgungssystem der Prävention und Frühintervention käme dem entgegen.
